# Characteristics and genetic analysis of patients suspected with early-onset systemic lupus erythematosus

**DOI:** 10.1186/s12969-022-00722-6

**Published:** 2022-08-13

**Authors:** Wan-Fang Lee, Wen-Lang Fan, Min-Hua Tseng, Huang-Yu Yang, Jing-Long Huang, Chao-Yi Wu

**Affiliations:** 1 Division of Allergy, Asthma, and Rheumatology, Department of Pediatrics, Chang Gung Memorial Hospital, No.5 Fu-Hsing St. Kuei Shan Hsiang, Taoyuan, Taoyuan Hsien Taiwan; 2 Genomic Medicine Research Core Laboratory, Chang Gung Memorial Hospital, Taoyuan, Taiwan; 3 College of Medicine, Chang Gung University, Taoyuan, Taiwan; 4 Division of Nephrology, Department of Pediatrics, Chang Gung Memorial Hospital, Taoyuan, Taiwan; 5 Department of Nephrology, Chang Gung Memorial Hospital, Taoyuan, Taiwan; 6 Department of Pediatrics, New Taipei Municipal TuCheng Hospital, New Taipei city, Taiwan; 7 Department of Medical Research, Kaohsiung Chang Gung Memorial Hospital, Kaohsiung, Taiwan

**Keywords:** Systemic lupus erythematous, Childhood lupus, Lupus mimics, Genetic study, TREX1, SLC7A7

## Abstract

**Background:**

Systemic lupus erythematosus (SLE) is rarely diagnosed before 5-years-old. Those with disease onset at a very young age are predicted by a higher genetic risk and a more severe phenotype. We performed whole-exome sequencing to survey the genetic etiologies and clinical manifestations in patients fulfilling 2012 SLICC SLE classification criteria before the age of 5.

**Case presentation:**

Among the 184 childhood-onset SLE patients regularly followed in a tertiary medical center in Taiwan, 7 cases (3.8%) of which onset ≦ 5 years of age were identified for characteristic review and genetic analysis. Compared to those onset at elder age, cases onset before the age of 5 are more likely to suffer from proliferative glomerulonephritis, renal thrombotic microangiopathy, neuropsychiatric disorder and failure to thrive. Causative genetic etiologies were identified in 3. In addition to the abundance of autoantibodies, patient with homozygous *TREX1* (c.292_293 ins A) mutation presented with chilblain-like skin lesions, peripheral spasticity, endocrinopathy and experienced multiple invasive infections. Patient with *SLC7A7* (c.625 + 1 G > A) mutation suffered from profound glomerulonephritis with full-house glomerular deposits as well as hyperammonemia, metabolic acidosis and episodic conscious disturbance. Two other cases harbored variants in lupus associating genes *C1s*, *C2*, *DNASE1* and *DNASE1L3* and another with *CFHR4.* Despite fulfilling the classification criteria for lupus, many of the patients required treatments beyond conventional therapy.

**Conclusions:**

Genetic etiologies and lupus mimickers were found among a substantial proportion of patients suspected with early-onset SLE. Detail clinical evaluation and genetic testing are important for tailored care and personalized treatment.

## Introduction

Systemic lupus erythematosus (SLE) is a complex autoimmune disease with heterogenous clinical manifestations. Characterized by immune dysregulation and production of autoantibodies against self-antigens, the disease can affect nearly any tissue or organ systems. To date, no diagnostic criteria for SLE is available [1]. To facilitate the suspicion of lupus and to compare between other autoimmune disorders, SLE classification criteria were established since 1972 and subsequently revised in 1982, 1997, 2012 and 2019 for refinement [2]. As the diagnosis of SLE mostly depend on clinical and serological clues, diagnosis of lupus can be challenging, especially among those with atypical manifestations and extreme phenotypes [1, 3].

Women of childbearing age are typically predisposed to developing SLE. Disease development before the age of 5 is relatively uncommon [4]. According to a nationwide study in Taiwan, the prevalence of SLE under the age of 5 was lower than 5/100,000 [5]. As genetic, hormonal and environmental factors are all known to contribute to disease development, through a large-scale multiracial SLE cohort study, Webb et al. found that the onset of lupus during childhood is predicted by a higher genetic risk and is associated with a more severe phenotype [6]. Moreover, considering the early-onset nature of “monogenic lupus” and some “SLE mimickers”, special attention and additional workup may be needed to assist the diagnosis of lupus in children before school age [7]. For decades, whole-exome sequencing (WES) and whole-genome sequencing have assisted in the identification of lupus-mimickers and rare monogenic variants associated with SLE with high penetrance [8]. Recognition of causative mutations in patients with early-onset lupus or lupus-mimics may provide crucial insights into the pathogenesis and improve personalized treatment [9].

To explore the clinical manifestations and causative mutations in patients suspected with early-onset SLE (age ≦ 5 years old) and lupus mimics in Taiwan, WES was performed with special attention on genes potentially responsible for lupus [10, 11]. Patient’s clinical features and treatment responses were also carefully reviewed.

## Patients and methods

### Study subjects

Between Jan. 2012 to Dec. 2019, one hundred and eighty-four childhood-onset SLE (cSLE) patients were regularly followed in the Pediatric Allergy, Asthma, and Rheumatology department in Chang-Gung Memorial Hospital, a tertiary medical center in Taiwan. The average age of disease onset was 12.9 ± 2.8 years old and 164 of them are female (89.1%). Among them, 7 cases (3.8%) of which onset ≦ 5 years of age were identified for detail characteristic review and genetic analysis. All cases fulfilled the 2012 Systemic Lupus International Collaborating Clinics (SLICC) criteria for the diagnosis of SLE [12].

### Genetic analysis

Genomic deoxyribonucleic acid (DNA) was isolated from peripheral venous blood samples. WES was performed at Biotools (New Taipei city, Taiwan) using the Agilent SureSelect Human All Exon Kit 58 m (v6) (Agilent Technologies, Inc. Santa Clara, United States) for exome capture and the NovaSeq 6000 platform (Illumina, San Diego, CA) for massively parallel sequencing. Raw image analyses and base calling were performed using Illumina’s Pipeline with default parameters. Sequence data were aligned to the reference human genome (hg38) using the Burrows-Wheeler Aligner, and duplicate reads were removed using Picard tools. Results revealed a mean depth of 62.34 times, and 98.38% of the targets were covered with at least 10 times of depths. We used the Genome Analysis Toolkit to perform realignment and variation (SNP and InDel) detection. Annovar was utilized to catalog the detected variations. Variations were filtered with a homo-polymer length > 6 (and synonymous substitutions) or that were common (> 1%) in the Exome Aggregation Consortium database and the Genome Aggregation Database. Pathogenicity score was calculated using PolyPhen2, SIFT, DANN and CADD. Special attention was placed on the panel of reported genes associating lupus to identify possible causal mutations [10, 11]. Sanger sequencing was performed to confirm the genetic variants from patient DNA and the parental DNA when the samples were available.

### Case presentation

Seven cases including 4 female (57.1%) and 3 male (42.9%) were suspected with early-onset SLE (age of onset ≦ 5 years old). None of the patients were of consanguineous marriage and the age of disease onset ranged from 20 to 60 months. Compared to the cSLE patients onset at elder age, cases fulfilling the SLE classification criteria before the age of 5 are less female dominate (57.1% vs 90.4%), and more likely to suffer from proliferative glomerulonephritis (71.4% vs 56.5%), renal thrombotic microangiopathy (TMA) (28.6% vs 4.5%), neuropsychiatric disorder (57.1% vs 11.3%), and failure to thrive (FTT) (42.9% vs 4.0%). Clinical manifestations and initial laboratorial findings of those suspected with early-onset SLE were summarized in Table 1. Patients with probable genetic etiologies were further discussed.

**Table 1:** Clinical characteristics and laboratory data of patients suspected with early-onset systemic lupus erythematosus

Cases	# 1	# 2	# 3	# 4	# 5	# 6	# 7
**Gender**	F	M	M	F	F	F	M
**Family history of SLE**	no	yes	yes	no	no	no	no
**Age when cSLE suspected (mon)**	20	24	38	48	51	60	60
**Age when WES performed (mon)**	21	27	46	50	101	96	103
**Identified genetic variants**	homozygous *TREX1* (c.292_293 ins A)	*SLC7A7* (c.625 + 1 G > A)	*SLC7A7* (c.625 + 1 G > A)	*DNASE1* (c.G370A)*,C1s* (c.G1241), *C2* (c.C1558T)	*CFHR4* (c.T103C)	*DNASE1L3* (c.G764A)	negative finding
**SLICC at diagnosis**							
Acute cutaneous lupus	-	-	-	-	-	+	-
Chronic cutaneous lupus	+	-	-	-	+	-	-
Oral or nasal ulcers	-	-	-	+	-	+	+
Nonscarring alopecia	-	-	-	-	-	-	-
Synovitis	-	-	-	-	+	+	+
Serositis	-	-	-	+	-	-	-
Renal	-	-	+ LN IV	+ LN IV	+ LN IV	+ LN IV	+ LN IV
Neurologic	+	-	-	-	-	-	-
Hemolytic anemia	+	-	-	+	+	-	-
Leukopenia/ lymphopenia	-	+	-	-	-	-	-
Thrombocytopenia	+	+	-	+	-	-	-
Positive ANA	+	-	+	+	+	+	+
Positive anti-dsDNA	-	+	+	-	+	+	+
Positive anti-Sm	+	NA	+	+	-	+	NA
Positive APL	+	NA	NA	+	-	-	-
Low complement level	-	+	+	+	+	+	+
Positive direct Coombs test	-	NA	-	-	-	+	+
**Initial Laboratory data**							
White blood cells (/μl) [RR: 4000–11000]	7600	4400	4300	4500	4400	7200	6000
Lymphocytes (/μl) [RR: 1000—4000]	1800	900	1500	2000	2200	2100	1500
Hemoglobin (g/dl) [RR: 12—18]	8.5	12.2	11.2	9.4	7.2	13	15.3
Platelets (K/μl) [RR: 150—400]	94	95	200	34	239	430	248
anti-dsDNA Ab (unit/ml) [RR: < 139]	104	140.5	176.6	49.5	714	615.5	> 2000
C3 (mg/dL) [RR: 90—180]	102	127	44.8	72	30.9	26.7	21.2
C4 (mg/dL) [RR: 10—40]	13.4	8.3	5.32	15.7	6.16	3.33	5.43
ANA [RR: 1:80 negative]	1:320	-	1:80	1:160	1:320	1:160	1:1280
Proteinuria [RR: 0]	0	1 +	4 +	4 +	2 +	4 +	2 +
Hematuria [RR: 0]	0	0	3 +	3 +	3 +	3 +	Trace
**Symptoms and comorbidities**							
Growth and development	FTT, developmental delay	-	FTT	-	FTT	-	-
CNS	conscious disturbance, encephalopathy with leukodystrophy	episodic conscious disturbance	episodic conscious disturbance	seizure with posterior reversible encephalopathy syndrome	-	-	-
Cardiovascular	patent foramen ovale, trivial tricuspid regurgitation	-	-	hypertension	hypertension	hypertension	hypertension
Gastrointestinal	gastroesophageal reflux, raise of hepatic enzyme	Hepatosplenomegaly, emesis	Hepatosplenomegaly, emesis	ascites	necrotizing pancreatitis with pancreatic pseudocysts	-	moderate hepatomegaly
Renal	-	-	GN	GN, TMA	GN, TMA	GN	GN
Metabolic	autoimmune thyroiditis with subclinical hypothyroidism, insulin-dependent diabetes mellitus	metabolic acidosis, hyperammonemia	metabolic acidosis, hyperammonemia	-	dyslipidemia	-	dyslipidemia
Mucocutaneous	chilblain-like skin rash	-	-	oral ulcer	discoid rash	oral ulcer, malar rash	oral ulcer
Musculoskeletal	Spastic quadriplegic cerebral palsy	-	delayed bone age	-	arthritis over bilateral knees	arthritis over bilateral ankles and knees	arthritis over bilateral knees
Serology	ANA, anti-Sm, anti-RNP, anti-Ro, anti-La, ANCA and APL Ab	anti-dsDNA, anti-Ro, low complement	ANA, anti-dsDNA, anti-Sm, and low complement	ANA, anti-Sm, APL and low complement	ANA, anti-dsDNA, anti-Ro and low complement	ANA, anti-dsDNA, anti-Sm, anti-Ro and low complement	ANA, anti-dsDNA, anti-Ro, APL and low complement
Invasive infections	recurrent aspiration pneumonia, P. aeruginosa pneumonia, Salmonella sepsis and K. pneumonia pyelonephritis	S. aureus bacteremia, M. catarrhalis pneumonia	-	-	Salmonella sepsis, C. albicans bacteremia, bacterial peritonitis	-	-
**Treatment**							
Prednisolone	tapering dose from 3.5 mg/day × 1 mon	tapering dose from 2.5 mg/day × 3 mons	tapering dose from 20 mg/day × 15 mons	tapering dose from 60 mg/day × 5 yrs	steroid pulse + tapering dose from 60 mg/day until now	steroid pulse + tapering dose from 60 mg/day until now	steroid pulse + tapering dose from 60 mg/day until now
Cyclophosphamide	-	-	-	-	0.5-1 g/m^2^ monthly × 5 doses	0.5-1 g/m^2^ monthly × 12 doses	0.5-1 g/m^2^ monthly × 12 doses
Azathioprine	-	-	-	-	-	-	50 mg/day × 2 mons
Cyclosporin	-	-	-	25–50 mg/day	100–150 mg/day	100–200 mg/day	-
MMF/MPA	-	-	-	MMF 500- 750 mg/day	MPA 360–1440 mg/day	MMF 500- 1500 mg/day	MPA 540–1440 mg/day
Hydroxychloroquine	-	-	-	-	100–200 mg/day	100–200 mg/day	100–200 mg/day
Other treatments	thyroxine, insulin	neomycin sulfate, sodium benzoate, multivitamins, lactulose	neomycin sulfate, sodium benzoate, multivitamins, lactulose	plasma exchange, captopril, amlodipine, clonidine	plasma exchange, captopril, losartan, atorvastatin	amlodipine	amlodipine, losartan

*Abbreviations*: *SLE* Systemic lupus erythematosus, *F* Female, *M* Male, *SLICC* Systemic Lupus International Collaborating Clinics, *LN IV* Lupus nephritis class 4, *ANA* Antinuclear antibody, *anti-dsDNA* Anti-double-stranded DNA antibody, *anti-Sm* Anti-Smith antibody, *anti-Ro* Anti-Ro antibody, *anti-La* Anti-La antibody, *ANCA* Anti-neutrophil cytoplasmic antibody, *anti-RNP* Anti-ribonucleoprotein antibody, *APL* Anti-phospholipid antibody, *CNS* Central nervous system, *TMA* Thrombotic microangiopathy, *GN* Glomerulonephritis, *MMF* Mycophenolate mofetil, *MPA* Mycophenolic acid, *NA* Not available, *RR* reference range

#### Case 1

**Fig. 1:**
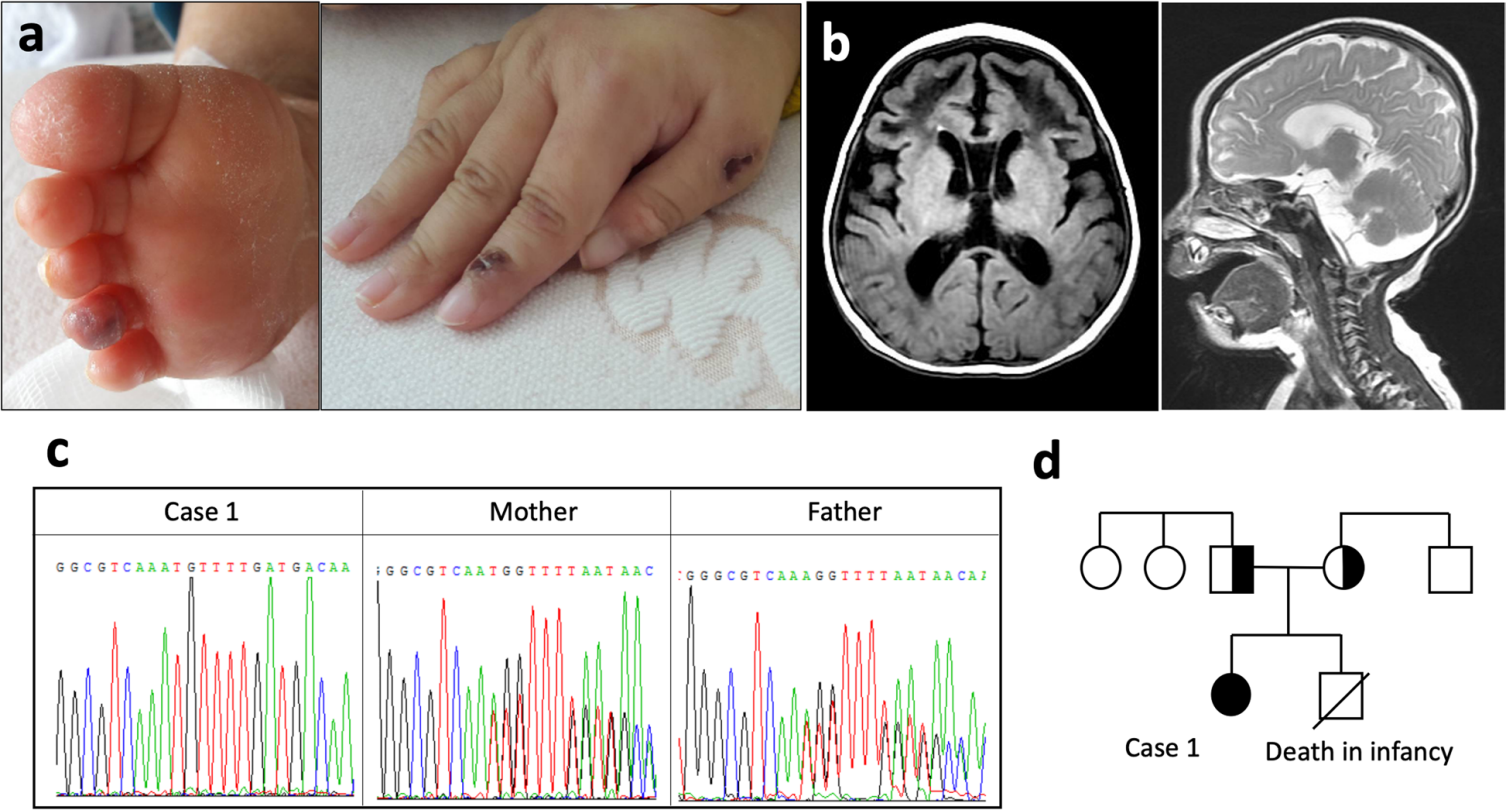
Clinical features and genetic analysis of Case 1. **a** Chilblain lupus erythematosus lesions over the ventral aspect of 4^th^ toe and dorsal aspect of the index finger and thumb. **b** The spectrum of brain changes, including encephalopathy with leukodystrophy on brain computed tomography. **c** Sanger sequencing of identified alterations with whole exome sequencing of a patient and her parents. **d** The family pedigree of case 1 with the *TREX1* mutation

Case 1 is a 20-month-old girl from a Taiwanese and Indonesian joint family presented with acute onset of drowsiness. On arrival, she was found with FTT, chilblain-like skin lesions and dystonic posturing with peripheral spasticity (Fig. 1A). Brain computed tomography imaging demonstrated encephalopathy with leukodystrophy (Fig. 1B**)**. Serial laboratory workup revealed thrombocytopenia, hemolytic anemia, positive anti-nuclear antibody (ANA) and high titer of anti-extractable nuclear antibodies (anti-ENA), including anti-Smith antibody (anti-Sm), anti-ribonucleoprotein antibody (anti-RNP), anti-Ro antibody, anti-La antibody, anti-neutrophil cytoplasmic antibody (ANCA) and anti-phospholipid antibody (APL) (Table 1). The patient was initially treated with low dose corticosteroids (~ 0.5 mg/kg/day) for progressing thrombocytopenia and hemolytic anemia, but quickly tapered off in a month due to limited response and aspiration pneumonia.

Because of her profound neurologic defects, chilblain-like skin rash and young age, WES and plasma interferon-α (IFN-α) was examined for type-1 interferonopathy survey. Her genetic analysis revealed homozygous *TREX1* c.292_293 ins A; p.Cys99Met fs mutation. The reported allelic frequency (AF) in gnomAD is 0.007% and the pathogenicity scores were unavailable. Sanger sequencing of the patient and her parental DNA confirmed that the *TREX1* variants were inherited from both of her parents. Level of her plasma IFN-α was significantly higher than both of her parents and health control (53.56 v.s. 38.91, 37.17 and 35.74 pg/ml, respectively). Under the diagnosis of Aicardi-Goutières syndrome (AGS), Janus kinase (JAK) inhibitor was suggested as an alternative choice but rapidly discontinued due to high expenses and recurrent infectious episodes. Autoimmune thyroiditis with subclinical hypothyroidism, glaucoma and insulin-dependent diabetes mellitus were noted during sequential follow-ups without steroid or immunosuppressants treatment. Despite careful care, the patient died at the age of 6 following multiple invasive infectious episodes, including recurrent aspiration pneumonia, Pseudomonas aeruginosa pneumonia, Salmonella sepsis and Klebsiella pneumonia pyelonephritis.

#### Case 2 and 3

**Fig. 2:**
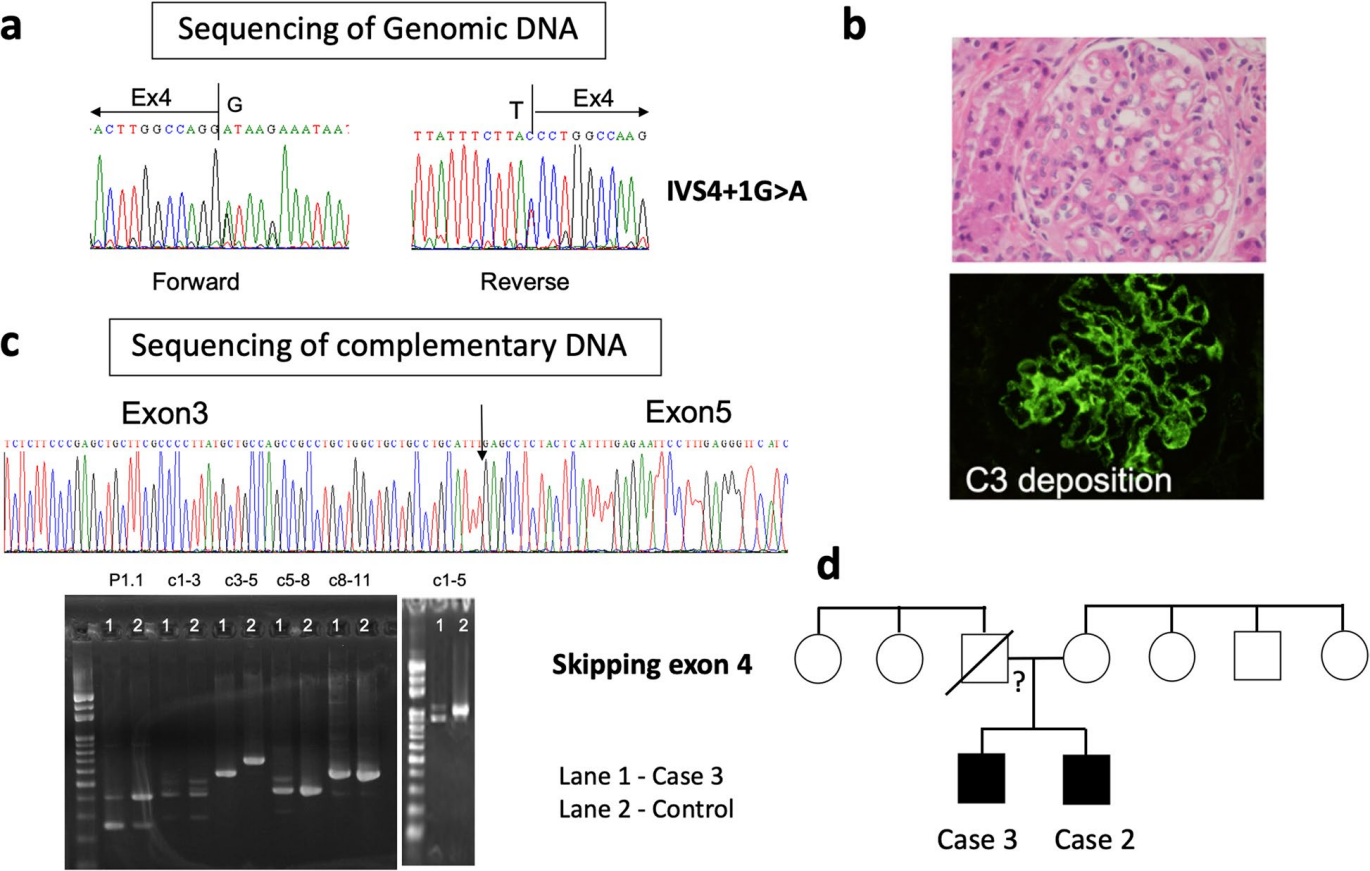
Renal histology and genetic/complementary DNA analysis of Case 2 & 3. **a** In the genomic level, the G before the last nucleotide of the intron is mutated to A, causing the splicing site to shift. **b** Histopathological examination of a renal biopsy showed membranoproliferative glomerulonephritis. An immunofluorescence micrograph illustrating diffuse glomerular C3 deposition. **c** Complementary DNA (cDNA) analysis revealed a skipping of exon 4. The band on gel electrophoresis confirmed a shorter cDNA product from the patient. **d** The family pedigree of Case 2 and 3 harboring heterozygous *SLC7A7* mutations. The father of Case 2 and 3 deceased and the mother is negative for *SLC7A7* mutation

In a nonconsanguineous family without documented family history of autoimmune diseases, 2 brothers sequentially fulfilled the 2012 SLICC SLE classification criteria at the ages of 24 and 38 months. Upon initial evaluation, the elder brother (case 3) suffered from FTT and glomerulonephritis presenting with profound proteinuria (> 1,000 mg/m^2^/day, urine protein/creatinine ratio: 98,198 mg/gm) and hematuria. Histopathological examination of his renal biopsy revealed membranoproliferative glomerulonephritis with strong IgG, IgA, IgM, C3 and C1q staining in diffuse pattern, compatible with lupus nephritis (LN) (Fig. 2B). Although his hemogram and clinical manifestations were unremarkable, positive ANA, anti-double stranded DNA antibody (anti-dsDNA), anti-Sm, low complements and the renal histopathological findings raised the suspicion of cSLE. Steroid (~ 2 mg/kg/day) was initially given for treatment and gradually tapered off in 15 months. His younger brother had a much milder symptoms with transient proteinuria, lymphopenia, thrombocytopenia, low C4 and positive autoantibody profile (Table 1). Episodic hyperammonemia, metabolic acidosis and conscious disturbance were noted during follow ups.

Due to young age, family cluster, metabolic disruption and conscious disturbance, genetic study was arranged. Share splicing variant c.625 + 1 G > A was found in the *SLC7A7* gene in both patients associating lysinuric protein intolerance (LPI), a rare metabolic disorder. The reported AF in gnomAD is 0.004% and the DANN and CADD pathogenicity score were 0.995 and 28, respectively. Although only one variant was identified by exome sequencing, sequencing of patient cDNA revealed a skipping of exon 4 compared to healthy controls for all transcripts (Fig. 2C). Sanger sequencing was not performed on the deceased father and the mother was negative for *SLC7A7* mutation (Fig. 2D). Instead of using immunosuppressants, these brothers were recommended to consume a hypoproteinemic diet adapted for their age and receive citrulline as well as vitamin supplementation. Case 2 experienced scattered episodes of hyperammonemia without serious conscious disturbance and the 2 brothers grew up smoothly without further immunomodulatory medications.

#### Case 4

Case 4 is a 4-year-old girl with painless oral ulceration and swelling legs. Seizure with posterior reversible encephalopathy syndrome was also noted. Renal biopsy following progressing edema and elevation of serum creatinine revealed LN class IV and renal TMA. Prednisone, mycophenolate mofetil (MMF) and plasma exchange were prescribed for rapid progressive glomerulonephritis. WES was arranged for young age and TMA. Her genetic analysis revealed heterozygous mutations in 3 different genes: *C1s* (c.G1241A; p.R414H; AF: 0.00478%; PolyPhen2: 0.096; DANN: 0.078), *C2* (c.C1558T; p.R520C; AF: 0.18%; PolyPhen2: 0.994; DANN: 0.999), and *DNASE1* (c.G370A; p.E124K; AF: 0.00521%; PolyPhen2: 0.687; DANN: 0.998). The patient is under relative stable condition under MMF and cyclosporin treatment.

#### Case 5

Case 5 is a 4-year-old girl with a record of FTT. Fever, arthritis, discoid rashes and high titer of autoantibodies were also noted (Table 1). Renal biopsy following heavy proteinuria, hematologic changes and hypertension revealed LN class IV with TMA. She underwent a course of plasma exchange due to persistent proteinuria despite cyclophosphamide, steroid and cyclosporine treatment. WES was arranged for young age and TMA. While no genetic variants among the SLE associating genes were found, a heterozygous mutation in the *CFHR4* gene (c.T103C; AF: 0.14%; PolyPhen2: 0.999; DANN: 0.952), was discovered. Deletions in the *CFHR4* gene have been found in association with atypical hemolytic uremic syndrome (aHUS), a form of TMA. Our patient suffered from recurrent aHUS and required plasma exchange, steroid, MMF and cyclosporine for treatment. Three episodes of necrotizing pancreatitis with pancreatic pseudocysts were noted 5 years after her diagnosis of SLE.

#### Case 6

Case 6 is a 5-year-old girl presented with nephrotic-range proteinuria (urine protein/creatinine ratio: 4,218 mg/gm), malar rash, oral ulceration and arthritis. Her renal biopsy suggested class IV LN. A heterozygous mutation in *DNASE1L3* (c.G764A; p.R255K; AF: 0.01%; PolyPhen2: 0.001; DANN: 0.705) was evidenced in her targeted WES screening. Treatment with cyclophosphamide pulse therapy, prednisolone, hydroxychloroquine and azathioprine lead to a complete remission of LN with improving proteinuria (urine protein/creatinine ratio: 38 mg/gm).

## Discussion

Clinical variation of SLE across different age groups existed in different populations [4, 13–17]. Utilizing the nationwide, population-based retrospective cohort, *Chen* et al. revealed that juvenile-onset SLE patients (onset < 18 years of age) were at greatest risk of mortality likely due to higher disease severity in Taiwan [16]. Systemic review and meta-analysis study performed by *Bundhun* et al. showed that renal inflammation, hematological manifestations, seizure and ocular involvement were significantly higher among young SLE patients [18]. In America, *Gomes* found no differences in gender discrepancy, nephritis, neuropsychiatric involvement and disease activity, but a higher frequency of fever, hepatomegaly, splenomegaly and discoid lupus among the early-onset SLE patients (onset < 6 years of age) [15]. Data collected from Europe suggested that young children with SLE have higher frequency of autoimmune family history, neuropsychiatric manifestations, nephritis, hematological disorders and an increased risk of organ damages potentially contributed by the cumulative duration and dose of prednisone and immunosuppressive medications [14, 17]. Similar but not limited to the reported findings, cases fulfilling the SLE classification criteria before the age of 5 in our cohort are less female dominate and more likely to suffer from proliferative glomerulonephritis, neuropsychiatric disorder as well as TMA and FTT. FTT is used to describe infant and child with weight below the fifth percentile for sex and corrected age [19]. While inadequate caloric intake was its leading etiology, inflammatory conditions, inborn errors of metabolism and genetic defects can all attributed its presence. The hallmarks of TMAs are vascular thromboses, which lead to clinical signs of microangiopathic hemolysis, a decrease in platelet count and organ damage involving renal or neurological manifestations [20]. It is a form of endothelial injury that can occur in the kidneys of 1–4% LN patients and is associated with severe clinical manifestations and a high mortality rate [21, 21]. Plasma exchange and Eculizumab, a monoclonal antibody capable of inhibiting C5 activation was recommended for treatment of TMA secondary to SLE [20, 21, 23]. Considering the necessity of further evaluation and treatment adjustment in the presence of FTT and TMA, it is worthwhile to look for these manifestations in cases suspected with early-onset SLE.

Two children with early-onset SLE, fulfilling the American College of Rheumatology (ACR) criteria for SLE was reported by *Hedrich* et al. with atypical manifestations including severe liver dysfunction, coagulopathy and protein loss enteropathy [4]. Through a large-scale multiracial SLE cohort study covering 1317 patients, *Webb* et al. discovered that the age of disease onset during childhood is predicted by a higher genetic risk for lupus and is associated with a more severe phenotype [6]. Recently, *Massias* et al. hypothesized that the variations of clinical manifestation across different age groups may resulted from different mechanisms underlining SLE pathophysiology at different age [13]. The availability of next generation sequencing and the emerging evidence of genetic susceptibility in lupus over the last decade largely expanded our knowledge on the genetic basis of lupus [8]. Mutations in genes governing the pathways of complement cascade, immune tolerance, apoptosis, antigen clearance, type I interferonopathy, metabolism and more have been identified as genetic etiologies underling lupus [10, 11, 24, 25]. Recently*, Tirosh* et al. reported that causative monogenic mutations were identified in 4 out of 15 newly diagnosed cSLE patients in 5 different genes: *C1QC, SLC7A7, MAN2B1, PTEN* and *STAT1* [11]. Analyzed 39 children with lupus manifestations associated with primary immunodeficiency diseases (PIDs), *Al-Mayouf* et al. discovered that complement deficiency was the most frequent PIDs associating lupus-like manifestations. Genetic defects in *PNP*, *PIK3CD, STAT1, ISG15, IL2RB, GS3, DNASE2* and genes associating chronic granulomatous disease were found among 7 of the 25 patients who underwent genetical testing [25]. Screening through 117 cSLE patients fulfilling the ACR criteria for SLE, *Belot* et al. reported that the mendelian genotypes involving variants in *C1QA, C1QC, C2, DNASE1L3*, and *IKZF1* were confirmed in 8 patients, while 7 additional cases harbored heterozygous variants in complement or type I interferon-associated autosomal recessive genes. Rare variants which were predicted to be damaging were significantly enriched in the cSLE cohort compared with controls [10]. In the present study, causative genetic etiologies *TREX1* and *SLC7A7* were identified in 3 out of 7 patients (42.9%). Rare and potentially damaging variants *C1s*, *C2*, *DNASE*, *DNASE1L3* and *CFHR4* were found in the other 3 (42.9%). Although monogenic lupus and lupus mimics were not exclusive for cases with early-onset disease, considering the high prevalence of causative genetic etiologies among the childhood population, detail clinical evaluation and genetic testing is recommended to help clarify the underling pathogenesis and predict disease course.

Abnormalities in the intracellular nucleic acid sensing machinery *TREX1* and other critical players including *RNASEH2A*, *RNASEH2B*, *RNASEH2C*, *SAMHD1*, *ADAR1* and *IFIH1* causes AGS, a monogenic interferonopathy [26, 27]. Due to the importance of type 1 interferon (IFN) in systemic autoimmune diseases, many overlapping clinical and laboratorial presentation were noted between patients with AGS and SLE. Genetic variant in *TREX1* gene is the causative mutation for Case 1. It encodes a 3’ repair exonuclease that guards DNA synthesis, and loss of function can lead to an accumulation of endogenous DNA and increased expression of IFN [28, 29]. Individuals with microcephaly or *TREX1-*related AGS, such as Case 1, were the most severely affected and less likely to achieve normal developmental milestone [26]. Although less discussed, besides the profound neurologic defects and presence of various autoantibodies, manifestations of congenital glaucoma [30], hepatic inflammation [31], endocrinopathies [32] and susceptible to infections [33] of Case 1 are also likely attributed to *TREX1* mutation. Worthwhile to mention, while the clinical features of heterozygous *TREX1* mutations have been described in patients with lupus [34, 35], the identification of homozygous *TREX1* (c.292_293 ins A) in SLE was reported for the first time. Comparing the age of disease onset in SLE mimics with monoallelic *TREX1* variants, the lupus phenotype appeared much earlier in Case 1 (20 month vs. 14 ~ 50 years old) [35]. Recently, JAK inhibitors including Ruxolitinib and Baricitinib have been reported to not only control AGS related skin lesions but neurologic function even in cases with severe and long-standing disease [36–38]. Other developing regimens, including interferon-α/β receptor blockade, IFN-α targeting, reverse-transcriptase inhibitors and stimulator of interferon genes (STING) antagonist also provide various degree of benefits as these medications suppress IFN signaling [39–44]. As targeted therapy became available, clinical suspicion and genetic testing for AGS is especially important for patients presenting with early-onset lupus mimic disease.

*SLC7A7* gene encoded a subunit of the cationic amino acid transporter found in epithelial cell membranes. Mutations in this gene causes LPI, a rare recessive disorder characterized by FTT, growth retardation, muscle hypotonia and hepatosplenomegaly [45]. Mostly appeared after weaning of breastmilk, clinical manifestations of LPI can be widely variable resembling the findings in urea cycle disorders such as hyperammonemia [46]. Overlapping manifestation of LPI and SLE has been reported in several case series [11, 46]. Renal involvement is a frequent and progressive complication in LPI. In a cohort of 39 LPI patients, 74% of the patients had proteinuria and 38% had hematuria [47]. Heterogeneous renal histological findings ranging from tubulointestinal disorder to distinct glomerulonephritis with polyclonal immunoglobulin deposition has been reported [47, 48]. Carefully reviewed by *Contreras* et al., the incidence of other LPI associating manifestations including FTT, metabolic disorder, neurologic symptoms and hepatosplenomegaly in Case 2 and 3 corresponded to 52%, 52%, 25% and 43% of all cases with LPI, respectively [46]. While recessive disorder requires homozygous *SLC7A7* defects to become phenotypic, the clinical phenotypes, presence of hyperammonemia, segregation analysis and the complete skipping of exon 4 revealed by complementary DNA analysis of Case 2 and 3 suggested a diagnosis of LPI. It is hypothesized that an undetected large deletion or compound heterozygous mutation existed in the corresponding allele, leading to the LPI and lupus phenotype. Instead of aggressive immunosuppressant treatment, administration of arginine and nitrogen scavenger drugs such as sodium benzoate and sodium phenylpyruvate to lower the level of ammonia and a low-protein diet with oral supplementation of citrulline and carnitine kept the 2 brothers under a relative stable disease status. In fact, dietary adjustment and nitrogen scavenger drugs are recommended as the mainstays of long-term therapy [49]. Identification of LPI, a lupus mimicker, from classical lupus thoroughly explained the atypical metabolic and neurological presentations among Case 2 and 3 and limited the use of unnecessary immunosuppressants. Moreover, the genetic data provided physicians with a better understanding on how the disease would progress. Potential complications including renal, hematological, skeletal, and gastrointestinal features will be closely monitored [49].

Rare and potentially pathogenic variants in the lupus associating genes *C1s*, *C2*, *DNASE1* and *DNASE1L3* as well as *CFHR4* were discovered in Cases 4–6 diagnosed with early-onset SLE in the present series. Homozygous deficiencies of early components within the complement cascades are among the strongest genetic risk factors for SLE in human [50]. SLE patient with *C1s* mutations has been reported with discoid rash, generalized seizure, autoimmune thyroiditis, autoimmune hepatitis and diffuse proliferative glomerulonephritis with full house deposition of glomerular immunofluorescence in their kidney biopsy consistent with LN [50–52]. Arthritis, mucocutaneous lesions, hematologic disorder and renal manifestations were documented among patients with *C2* mutations [53–55]. *DNASE1* mutation is associated with high titer of ANA, anti-dsDNA, anti-histones, anti-Ro and immune mediated glomerulonephritis [56]. Interestingly, although the direct contribution of these mutations in Case 4 presenting with various autoantibodies, proliferative LN and seizure associating posterior reversible encephalopathy syndrome remain unknown, reduced plasma DNase1 activity have recently been shown to cause the persistence of pro-thrombotic neutrophil extracellular traps, promote microvascular thrombosis and contribute the development of TMA [57]. Patients with *DNASE1L3* mutations are prone for LN, high titer of ANA, APL, ANCA and low complement similar to Case 6 [58]. *CFHR4* encodes one of the 5 complement factor H-related proteins and is linked to aHUS, a life threatening TMA characterized by dysregulation of the alternative pathway of complement [59]. While aHUS rarely causes acute pancreatitis and the association between these two diseases remain unclear, several reports revealed an episode of acute pancreatitis preceding TMA in cases with or without TMA related mutations [60–62]. Together, although the direct impact of these variants in disease manifestation requires further clarification, the accumulation of rare variants predicted-damaging variants in SLE-associated genes may contribute to disease expression and clinical heterogeneity [10].

Considering the prevalence and the severity of proliferative glomerulonephritis, TMA and neuropsychiatric disorder in cases suspected with early-onset SLE, identification of genetic variants associating these phenotypes may be as important as surveying for lupus associating mutations in young children with lupus-like manifestations. CNS manifestations of SLE expanded widely from nonspecific symptoms including headache, cognitive impairment to devastating features such as memory loss, seizures and stroke [63]. As previously reviewed, only handful of genetic variations were associated with neuropsychiatric symptoms in lupus [63, 64]. The HLA-DRB1Ã04 genotype and *STAT4* rs10181656 were associated with stroke in SLE independent of the status of APL [65, 66]. Rare single-nucleotide polymorphisms (SNPs) and mutations in *TREX1*, have been reported in SLE cases with neurological manifestations, especially seizures and neuropsychiatric lupus [35, 66, 67]. The cumulative effect of having 10 or more SNPs in the *HLADRB1, IRF5, STAT4, BLK, TNFAIP3, TNIP1, FCGR2B* and *TNFSF13* genes have also been shown to increase the risk of developing neurological manifestations by twofold [68]. Changes in mental status were noted in 3 of the 7 patients in the present series. While delirium, depression, dementia, and coma can all result in mental status alteration, *TREX1* associated encephalopathy affecting the ascending reticular activating system and LPI related hyperammonemia and metabolic acidosis possibly attributed to their neuropsychiatric presentation. Proliferative glomerulonephritis can lead to end-stage kidney disease and usually requires aggressive treatment with immunosuppressants [69]. Recently, around 60 different disease susceptibility genes associated with LN were classified according to the pathways they’re involved [70]. Take *BLK* mutation for example, being a src family non-receptor tyrosine kinase mainly expressed by B-cells, *BLK* mutations in LN not only interrupt one’s adaptive immune signaling, but provide a rational for the application of B cell targeting regimen in the control of LN [70]. *DNASE1* mutation in Case 4 alters program cell death and *TREX1* mutation in Case 1 mainly affects the innate immunity. Despite the notion of its potential functionality, no pathway-specific therapeutic strategies, however, were recommended for LN related to these mutations. Finally, genetic mutations including *CFH*, *CFI*, *CFB*, C3, *THBD, PLG*, *MCP, ADAMTS13, MMACHC* and *DGKE* have recently been reported to result in TMA [20, 23, 71]. Spotting a genetic variant in the TMA associating genes by physician should raise the awareness of TMA in patients suspected with early-onset lupus.

During the past decades, treatment for SLE has moved from corticosteroids alone to a combination of disease-modifying antirheumatic drugs, immunosuppressants and biologics. A treat-to-target strategy was recently proposed for lupus, leading to individualized, patient-tailored regimens with multitargeted therapies [9, 72]. With rapid expansion of treatment options, early identification of patients with lupus mimics and those with causative mutations from sporadic lupus is necessary for precise and tailored treatment. Recently, it is proposed that genetic testing are recommended for those with disease onset at a young age; severe, life-threatening or organ-threatening presentation; aggressive disease course, rapid deterioration and/or accumulation of organ damage; and poor response to standard treatment [11]. Due to the rarity of early-onset SLE, we are unable to enroll large enough case number in the study to reflect the clinical significance of individualized treatment. International and multi-central collaboration is needed to better address the issue. In the coming era of precision medicine, patients with SLE will likely be stratified by their immunophenotypes or their genetics as technology advances [9, 72]. Revealing the molecular genetic diagnosis of SLE, especially among those early-onset cases, can promote personalized medical care with targeted therapies and monitoring.

## Conclusions

Genetic etiologies and lupus mimickers were found among a substantial proportion of patients suspected with early-onset SLE. Detail clinical evaluation and genetic testing are important to predict disease course, organ damages and refine the therapeutic options for pathology-based precision medicine.

## Data Availability

The datasets used and/or analyzed during the current study are available from the corresponding author on reasonable request.

## Abbreviations

ACR: American College of Rheumatology
AF: Allelic frequency
AGS: Aicardi-Goutières syndrome
aHUS: Atypical hemolytic uremic syndrome
Anti-dsDNA: Anti-double stranded DNA antibody
anti-ENA: Anti-extractable nuclear antibody
Anti-La: anti-La antibody
ANA: Anti-nuclear antibody
ANCA: Anti-neutrophil cytoplasmic antibody
APL: Anti-phospholipid antibody
anti-RNP: Anti-ribonucleoprotein antibody
Anti-Ro: anti-Ro antibody
anti-Sm: Anti-Smith antibody
cSLE: Childhood-onset SLE
CNS: Central nervous system
DNA: Deoxyribonucleic acid
FTT: Failure to thrive
gnomAD: Genome Aggregation Database
IFN: Interferon
JAK: Janus kinase
LPI: Lysinuric protein intolerance
LN: Lupus nephritis
MMF: Mycophenolate mofetil
MPA: Mycophenolic acid
PID: Primary immunodeficiency
SLE: Systemic lupus erythematosus
SLICC: Systemic Lupus International Collaborating Clinics
SNP: Single nucleotide polymorphism
TMA: Thrombotic microangiopathy
WES: Whole-exome sequencing

## References

[1] Fanouriakis A, Tziolos N, Bertsias G, Boumpas DT (2021). Update omicronn the diagnosis and management of systemic lupus erythematosus. Ann Rheum Dis.

[2] Aringer M, Costenbader K, Daikh D, Brinks R, Mosca M, Ramsey-Goldman R (2019). 2019 European league against rheumatism/american college of rheumatology classification criteria for systemic lupus erythematosus. Ann Rheum Dis.

[3] Chasset F, Richez C, Martin T, Belot A, Korganow AS, Arnaud L (2019). Rare diseases that mimic systemic lupus erythematosus (lupus mimickers). Joint Bone Spine.

[4] Hedrich CM, Zappel H, Straub S, Laass MW, Wieczorek K, Hahn G (2011). Early onset systemic lupus erythematosus: differential diagnoses, clinical presentation, and treatment options. Clin Rheumatol.

[5] Huang JL, Yao TC, See LC (2004). Prevalence of pediatric systemic lupus erythematosus and juvenile chronic arthritis in a Chinese population- a nation-wide prospective population-based study in Taiwan. Clin Exp Rheumatol.

[6] Webb R, Kelly JA, Somers EC, Hughes T, Kaufman KM, Sanchez E (2011). Early disease onset is predicted by a higher genetic risk for lupus and is associated with a more severe phenotype in lupus patients. Ann Rheum Dis.

[7] Alperin JM, Ortiz-Fernandez L, Sawalha AH (2018). Monogenic lupus: a developing paradigm of disease. Front Immunol.

[8] Chung SA, Shum AK (2016). Rare variants, autoimmune disease, and arthritis. Curr Opin Rheumatol.

[9] Demirkaya E, Sahin S, Romano M, Zhou Q, Aksentijevich I (2020). New horizons in the genetic etiology of systemic lupus erythematosus and lupus-like disease: monogenic lupus and beyond. J Clin Med..

[10] Belot A, Rice GI, Omarjee SO, Rouchon Q, Smith EMD, Moreews M (2020). Contribution of rare and predicted pathogenic gene variants to childhood-onset lupus: a large, genetic panel analysis of British and French cohorts. The Lancet Rheumatology.

[11] Tirosh I, Spielman S, Barel O, Ram R, Stauber T, Paret G (2019). Whole exome sequencing in childhood-onset lupus frequently detects single gene etiologies. Pediatr Rheumatol Online J.

[12] Petri M, Orbai AM, Alarcon GS, Gordon C, Merrill JT, Fortin PR (2012). Derivation and validation of the systemic lupus international collaborating clinics classification criteria for systemic lupus erythematosus. Arthritis Rheum.

[13] Massias JS, Smith EMD, Al-Abadi E, Armon K, Bailey K, Ciurtin C (2020). Clinical and laboratory characteristics in juvenile-onset systemic lupus erythematosus across age groups. Lupus.

[14] Fonseca R, Aguiar F, Rodrigues M, Brito I (2018). Clinical phenotype and outcome in lupus according to age: a comparison between juvenile and adult onset. Reumatol Clin (Engl Ed).

[15] Gomes RC, Silva MF, Kozu K, Bonfa E, Pereira RM, Terreri MT (2016). Features of 847 childhood-onset systemic lupus erythematosus patients in three age groups at diagnosis: a Brazilian multicenter study. Arthritis Care Res (Hoboken).

[16] Chen YM, Lin CH, Chen HH, Chang SN, Hsieh TY, Hung WT (2014). Onset age affects mortality and renal outcome of female systemic lupus erythematosus patients: a nationwide population-based study in Taiwan. Rheumatology (Oxford).

[17] Descloux E, Durieu I, Cochat P, Vital-Durand D, Ninet J, Fabien N (2009). Influence of age at disease onset in the outcome of paediatric systemic lupus erythematosus. Rheumatology (Oxford).

[18] Bundhun PK, Kumari A, Huang F (2017). Differences in clinical features observed between childhood-onset versus adult-onset systemic lupus erythematosus: a systematic review and meta-analysis. Medicine (Baltimore).

[19] Yoo SD, Hwang EH, Lee YJ, Park JH (2013). Clinical Characteristics of Failure to Thrive in Infant and Toddler: Organic vs. Nonorganic Pediatr Gastroenterol Hepatol Nutr.

[20] Aigner C, Schmidt A, Gaggl M, Sunder-Plassmann G (2019). An updated classification of thrombotic microangiopathies and treatment of complement gene variant-mediated thrombotic microangiopathy. Clin Kidney J.

[21] Wright RD, Bannerman F, Beresford MW, Oni L (2020). A systematic review of the role of eculizumab in systemic lupus erythematosus-associated thrombotic microangiopathy. BMC Nephrol.

[22] Lansigan F, Isufi I, Tagoe CE (2010). Microangiopathic haemolytic anaemia resembling thrombotic thrombocytopenic purpura in systemic lupus erythematosus: the role of ADAMTS13. Rheumatology.

[23] Kello N, Khoury LE, Marder G, Furie R, Zapantis E, Horowitz DL (2019). Secondary thrombotic microangiopathy in systemic lupus erythematosus and antiphospholipid syndrome, the role of complement and use of eculizumab: Case series and review of literature. Semin Arthritis Rheum.

[24] Costa-Reis P, Sullivan KE (2017). Monogenic lupus: it’s all new!. Curr Opin Immunol.

[25] Al-Mayouf SM, Alreefi HA, Alsinan TA, AlSalmi G, AlRowais A, Al-Herz W (2021). Lupus manifestations in children with primary immunodeficiency diseases: comprehensive phenotypic and genetic features and outcome. Mod Rheumatol.

[26] Adang L, Gavazzi F, De Simone M, Fazzi E, Galli J, Koh J (2020). Developmental outcomes of aicardi goutieres syndrome. J Child Neurol.

[27] Crow YJ, Stetson DB. The type I interferonopathies: 10 years on. Nat Rev Immunol. 2022;22(8):471-83. 10.1038/s41577-021-00633-9 PMC8527296 34671122

[28] Grieves JL, Fye JM, Harvey S, Grayson JM, Hollis T, Perrino FW (2015). Exonuclease TREX1 degrades double-stranded DNA to prevent spontaneous lupus-like inflammatory disease. Proc Natl Acad Sci U S A.

[29] Rice GI, Rodero MP, Crow YJ (2015). Human disease phenotypes associated with mutations in TREX1. J Clin Immunol.

[30] Gowda VK, Vegda H, Shivappa SK, Benakappa N (2020). Aicardi-goutieres syndrome presenting with congenital glaucoma. Indian J Pediatr.

[31] Gavazzi F, Cross ZM, Woidill S, McMann JM, Rand EB, Takanohashi A (2021). Hepatic involvement in aicardi-goutieres syndrome. Neuropediatrics.

[32] Worth C, Briggs TA, Padidela R, Balmer E, Skae M (2020). Endocrinopathies in aicardi goutieres syndrome-a descriptive case series. Clin Case Rep.

[33] Li P, Du J, Goodier JL, Hou J, Kang J, Kazazian HH (2017). Aicardi-Goutieres syndrome protein TREX1 suppresses L1 and maintains genome integrity through exonuclease-independent ORF1p depletion. Nucleic Acids Res.

[34] Lee-Kirsch MA, Gong M, Chowdhury D, Senenko L, Engel K, Lee YA (2007). Mutations in the gene encoding the 3’-5’ DNA exonuclease TREX1 are associated with systemic lupus erythematosus. Nat Genet.

[35] Namjou B, Kothari PH, Kelly JA, Glenn SB, Ojwang JO, Adler A (2011). Evaluation of the TREX1 gene in a large multi-ancestral lupus cohort. Genes Immun.

[36] Chyuan IT, Tzeng H-T, Chen J-Y (2019). Signaling Pathways of Type I and Type III Interferons and Targeted Therapies in Systemic Lupus Erythematosus. Cells.

[37] Briand C, Fremond ML, Bessis D, Carbasse A, Rice GI, Bondet V (2019). Efficacy of JAK1/2 inhibition in the treatment of chilblain lupus due to TREX1 deficiency. Ann Rheum Dis.

[38] Vanderver A, Adang L, Gavazzi F, McDonald K, Helman G, Frank DB (2020). Janus kinase inhibition in the aicardi-goutieres syndrome. N Engl J Med.

[39] Ronnblom L, Leonard D (2019). Interferon pathway in SLE: one key to unlocking the mystery of the disease. Lupus Sci Med.

[40] Hong Z, Mei J, Li C, Bai G, Maimaiti M, Hu H (2021). STING inhibitors target the cyclic dinucleotide binding pocket. Proc Natl Acad Sci U S A..

[41] Rice GI (2018). Reverse-transcriptase inhibitors in the aicardi-goutières syndrome. N Engl J Med..

[42] Crow YJ, Shetty J, Livingston JH (2019). Treatments in Aicardi-Goutières syndrome. Dev Med Child Neurol.

[43] Rice GI, Meyzer C, Bouazza N, Hully M, Boddaert N, Semeraro M (2018). Reverse-transcriptase inhibitors in the aicardi-goutieres syndrome. N Engl J Med.

[44] Mura E, Masnada S, Antonello C, Parazzini C, Izzo G, Garau J (2021). Ruxolitinib in aicardi-goutieres syndrome. Metab Brain Dis.

[45] Torrents D, Mykkänen J, Pineda M, Feliubadaló L, Estévez R, Cid RD (1999). Identification of SLC7A7, encoding y+LAT-1, as the lysinuric protein intolerance gene. Nature Genetics..

[46] Contreras JL, Ladino MA, Aranguiz K, Mendez GP, Coban-Akdemir Z, Yuan B (2021). Immune dysregulation mimicking systemic lupus erythematosus in a patient with lysinuric protein intolerance: case report and review of the literature. Front Pediatr.

[47] Nicolas C, Bednarek N, Vuiblet V, Boyer O, Brassier A, De Lonlay P (2016). Renal involvement in a french paediatric cohort of patients with lysinuric protein intolerance. JIMD Rep.

[48] Esteve E, Krug P, Hummel A, Arnoux JB, Boyer O, Brassier A (2017). Renal involvement in lysinuric protein intolerance: contribution of pathology to assessment of heterogeneity of renal lesions. Hum Pathol.

[49] Noguchi A, Takahashi T (2019). Overview of symptoms and treatment for lysinuric protein intolerance. J Hum Genet.

[50] Wu YL, Brookshire BP, Verani RR, Arnett FC, Yu CY (2011). Clinical presentations and molecular basis of complement C1r deficiency in a male African-American patient with systemic lupus erythematosus. Lupus.

[51] Amano MT, Ferriani VPL, Florido MPC, Reis ES, Delcolli MIMV, Azzolini AECS (2008). Genetic analysis of complement C1s deficiency associated with systemic lupus erythematosus highlights alternative splicing of normal C1s gene. Mol Immunol.

[52] Batu ED, Koşukcu C, Taşkıran E, Sahin S, Akman S, Sözeri B (2018). Whole exome sequencing in early-onset systemic lupus erythematosus. J Rheumatol.

[53] Liphaus BL, Umetsu N, Jesus AA, Bando SY, Silva CA, Carneiro-Sampaio M (2015). Molecular characterization of the complement C1q, C2 and C4 genes in Brazilian patients with juvenile systemic lupus erythematosus. Clinics.

[54] Jönsson G, Sjöholm AG, Truedsson L, Bengtsson AA, Braconier JH, Sturfelt G (2007). Rheumatological manifestations, organ damage and autoimmunity in hereditary C2 deficiency. Rheumatology.

[55] Jönsson G, Truedsson L, Sturfelt G, Oxelius V-A, Braconier JH, Sjöholm AG (2005). Hereditary C2 deficiency in Sweden. Medicine.

[56] Felux J, Erbacher A, Breckler M, Herve R, Lemeiter D, Mannherz HG (2021). Deoxyribonuclease 1-mediated clearance of circulating chromatin prevents from immune cell activation and pro-inflammatory cytokine production, a phenomenon amplified by low Trap1 activity: consequences for systemic lupus erythematosus. Front Immunol.

[57] Jimenez-Alcazar M, Napirei M, Panda R, Kohler EC, Kremer Hovinga JA, Mannherz HG (2015). Impaired DNase1-mediated degradation of neutrophil extracellular traps is associated with acute thrombotic microangiopathies. J Thromb Haemost.

[58] Al-Mayouf SM, Sunker A, Abdwani R, Abrawi SA, Almurshedi F, Alhashmi N (2011). Loss-of-function variant in DNASE1L3 causes a familial form of systemic lupus erythematosus. Nat Genet.

[59] Martin Merinero H, Zhang Y, Arjona E, Del Angel G, Goodfellow R, Gomez-Rubio E (2021). Functional characterization of 105 factor H variants associated with aHUS: lessons for variant classification. Blood.

[60] Beck BB, van Spronsen F, Diepstra A, Berger RM, Komhoff M (2017). Renal thrombotic microangiopathy in patients with cblC defect: review of an under-recognized entity. Pediatr Nephrol.

[61] Jean-Marie EM, Cho JJ, Trevino JG (2020). A case report of recurrent acute pancreatitis associated with life threatening atypical hemolytic uremic syndrome. Medicine (Baltimore).

[62] Sandino-Perez J, Gutierrez E, Caravaca-Fontan F, Morales E, Aubert-Girbal L, Delgado-Lillo R (2021). Haemolytic uraemic syndrome associated with pancreatitis: report of four cases and review of the literature. Clin Kidney J.

[63] Schwartz N, Stock AD, Putterman C (2019). Neuropsychiatric lupus: new mechanistic insights and future treatment directions. Nat Rev Rheumatol.

[64] Fanouriakis A, Boumpas DT, Bertsias GK (2013). Pathogenesis and treatment of CNS lupus. Curr Opin Rheumatol.

[65] Lundstrom E, Gustafsson JT, Jonsen A, Leonard D, Zickert A, Elvin K (2013). HLA-DRB1*04/*13 alleles are associated with vascular disease and antiphospholipid antibodies in systemic lupus erythematosus. Ann Rheum Dis.

[66] Rullo OJ, Tsao BP (2013). Recent insights into the genetic basis of systemic lupus erythematosus. Ann Rheum Dis..

[67] Ceccarelli F, Perricone C, Borgiani P, Ciccacci C, Rufini S, Cipriano E (2015). genetic factors in systemic lupus erythematosus: contribution to disease phenotype. J Immunol Res.

[68] Koga M, Kawasaki A, Ito I, Furuya T, Ohashi J, Kyogoku C (2011). Cumulative association of eight susceptibility genes with systemic lupus erythematosus in a Japanese female population. J Hum Genet.

[69] Wu JY, Yeh KW, Huang JL (2014). Early predictors of outcomes in pediatric lupus nephritis: focus on proliferative lesions. Semin Arthritis Rheum.

[70] Munroe ME, James JA (2015). Genetics of lupus nephritis: clinical implications. Semin Nephrol.

[71] Vieira-Martins P, El Sissy C, Bordereau P, Gruber A, Rosain J, Fremeaux-Bacchi V (2016). Defining the genetics of thrombotic microangiopathies. Transfus Apheres Sci.

[72] Nagafuchi Y, Shoda H, Fujio K (2019). Immune Profiling and Precision Medicine in Systemic Lupus Erythematosus. Cells..

